# Hydrogen dangling bonds induce ferromagnetism in two-dimensional metal-free graphitic-C_3_N_4_ nanosheets†
†Electronic supplementary information (ESI) available: Experimental and characterization. See DOI: 10.1039/c4sc02576h


**DOI:** 10.1039/c4sc02576h

**Published:** 2014-10-01

**Authors:** Kun Xu, Xiuling Li, Pengzuo Chen, Dan Zhou, Changzheng Wu, Yuqiao Guo, Lidong Zhang, Jiyin Zhao, Xiaojun Wu, Yi Xie

**Affiliations:** a Hefei National Laboratory for Physical Sciences at Microscale , University of Science and Technology of China , Hefei , P. R. China . Email: czwu@ustc.edu.cn; b CAS Key Laboratory of Materials for Energy Conversion , Department of Material Science and Engineering , University of Science and Technology of China , Hefei , P. R. China; c National Synchrotron Radiation Laboratory , University of Science and Technology of China , Hefei , P. R. China

## Abstract

Ferromagnetic two-dimensional (2D) ultrathin nanosheets hold great promise for next generation electronics. Ferromagnetic metal-free materials that usually possess only an s/p electronic configuration with weak spin–orbit coupling and a large spin relaxation time, would play an important role in constructing future spintronic devices. However, the absence of an intrinsic spin ordering structure in most metal-free materials greatly hampers the widening scope of ferromagnetic 2D nanostructures as well as in-depth understanding of their ferromagnetic nature. Herein, the induction of intrinsic ferromagnetism in 2D metal-free g-C_3_N_4_ ultrathin nanosheets has been achieved through a new effective strategy whereby hydrogen dangling bonds are introduced. In our case, g-C_3_N_4_ ultrathin nanosheets with hydrogen dangling bonds showed obvious room temperature ferromagnetic behavior that could even be tuned by the concentration of hydrogen. This work will pave a new pathway to engineer the properties of 2D nanomaterial systems.

## Abstract 2

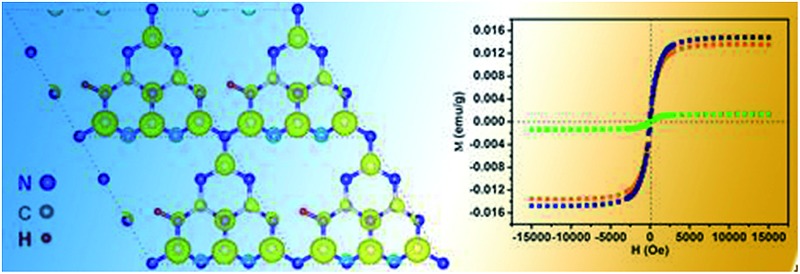
Inducing ferromagnetism in two dimensional metal-free g-C_3_N_4_ nanosheets has been achieved through the introduction of hydrogen dangling bonds.

## Introduction

Two dimensional (2D) ultrathin nanosheets are of great interest in bringing about exotic physical properties arising from their dimensional reduction systems, and hold enormous promise for next generation electronic devices.^1–3^ Recent investigations of ferromagnetic 2D nanomaterials with unique spin ordering have received extensive attention due to their potential application in future spintronic devices.^4,5^ However, although significant efforts have been made to develop 2D ferromagnetic nanosheets with a 3d electronic configuration,^6–8^ ferromagnetic 2D metal-free ultrathin nanosheets with only an s/p electronic structure present relatively weak spin–orbit coupling and could give a large spin relaxation time that are more suitable for future-generation spintronic devices.^9^ Unfortunately, metal-free materials usually lack an ordered spin structure in their pristine forms, greatly hampering the appearance of ferromagnetic behavior in their 2D nanostructures. Thus, it is highly desirable to achieve magnetic coupling modulation in 2D metal-free ultrathin nanosheets for future-generation spintronic devices.

Hydrogenation provides a new intriguing strategy to regulate the electronic structure of materials, endowing a new opportunity to induce spin related information in 2D metal-free ultrathin nanosheets.^10,11^ Serial theoretical calculations have already predicated that hydrogenation of graphene could induce ferromagnetic behavior.^12,13^ In this case, hydrogenation of graphene, *i.e.* modifying hydrogen atoms onto the in-plane surface carbon atoms, would be extremely experimentally challenging because the configuration change from sp^2^ to sp^3^ hybridization requires overcoming a considerable energy barrier.^14^ However, graphite carbon nitride (g-C_3_N_4_), a graphite-like material with C–N layers in a weakly stacked structure, shows advancements for hydrogenation.^15,16^ For g-C_3_N_4_, there is only one non-bonding electron in the C atom with the sp^2^ hybridized structure, while lone pair electrons or more electrons would be remnant in the N atom with the sp^3^ or sp^2^ hybridized structure. In this regard, manipulating a hydrogen dangling bond interaction in g-C_3_N_4_ ultrathin nanosheets is much easier than that in graphene from an experimental viewpoint. In effect, g-C_3_N_4_ has attracted tremendous attention due to its unique electronic band structure catering for intriguing applications in catalysis, sensing, bioimaging and so on.^17–20^ Thus, it remains an open question whether hydrogen dangling bonds could endow spin-related information in a graphene-like g-C_3_N_4_ structure.

**Fig. 1:**
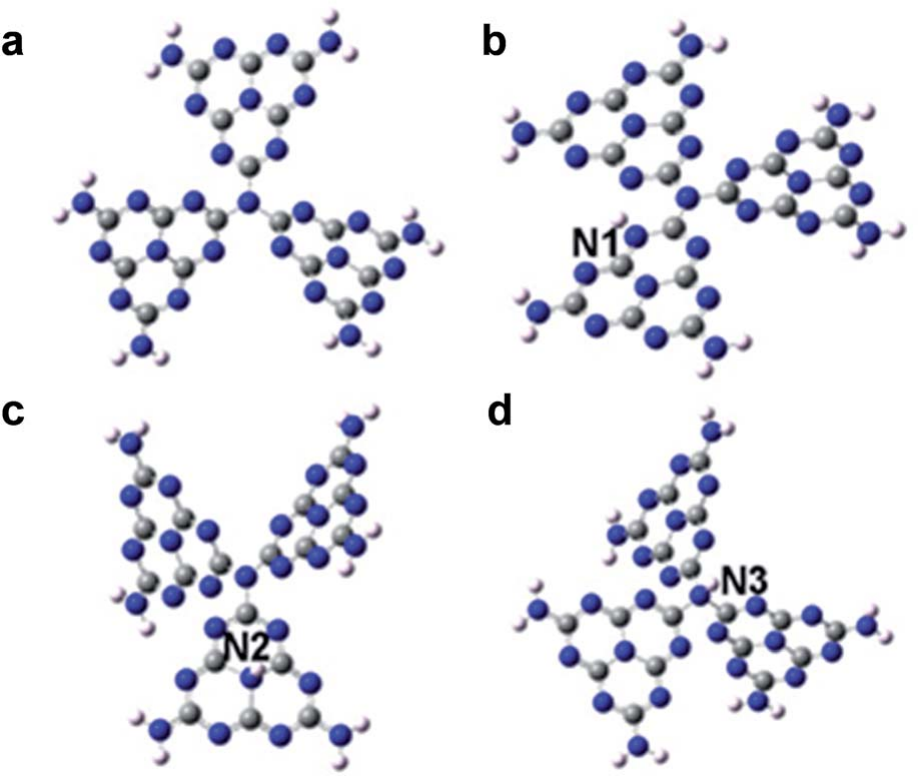
The calculated molecular models of (a) g-C_3_N_4_, (b) g-C_3_N_4_ with hydrogen dangling bonds in the N1 site, (c) with hydrogen dangling bonds in the N2 site, and (d) with hydrogen dangling bonds in the N3 site. Gray, blue and white balls represent C, N and H atoms, respectively.

Density-functional theory based on the g-C_3_N_4_ structure model consisting of 3 tris-*s*-triazine (melem) units revealed that hydrogen dangling bonds in N2 sites would not bring spin-related information. Interestingly, the hydrogen bonds in the N1 and N3 sites could induce ferromagnetism. Further theoretical calculations were carried out with the M062X/6-31G(d,p) method in the Gaussian 09 program package to study the total energy of the hydrogen dangling bonds in different N sites of the g-C_3_N_4_ structure (Fig. 1).^21,22^ The hydrogen bonds in the N1 sites are more stable than those in the N2 and N3 sites, with energy differences of 43.06 kcal mol^–1^ and 38.96 kcal mol^–1^, respectively. Based on the analysis above, engineering the hydrogen dangling bonds in the g-C_3_N_4_ ultrathin nanosheets shows a promising sign to induce intrinsic ferromagnetism in 2D metal-free nanomaterials. Herein, a new room temperature ferromagnetic 2D nanomaterial, g-C_3_N_4_ ultrathin nanosheets with hydrogen dangling bonds, was confirmed for the first time. In this case, the spin ordering structure was endowed in the 2D g-C_3_N_4_ ultrathin nanosheets by introducing the hydrogen dangling bonds. Interestingly, the saturation magnetization of g-C_3_N_4_ could even be tuned by increasing the hydrogen dangling bond content. The hydrogen dangling bonds in the g-C_3_N_4_ ultrathin nanosheets brought an impressive saturation magnetization value of 0.015 emu g^–1^, associated with a coercivity of 87 Oe at room temperature. To the best of our knowledge, it is the first experimental case showing that hydrogen dangling bonds could achieve magnetic coupling modulated in 2D metal-free g-C_3_N_4_ systems.

## Results and discussion

**Fig. 2:**
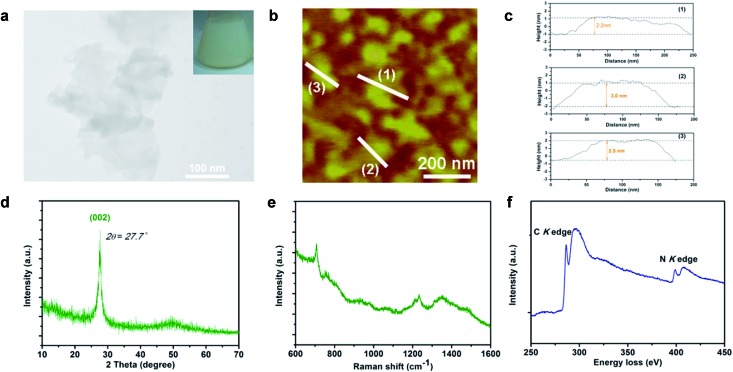
Characterization of CN-4. (a) TEM image. Inset: homogeneously dispersed CN-4 nanosheet suspension. (b) and (c) AFM image and the corresponding height profile. (d) XRD pattern. (e) Raman spectrum. (f) EELS spectrum.

G-C_3_N_4_ ultrathin nanosheets with hydrogen dangling bonds (denoted as CN-3) are obtained by liquid exfoliation of bulk g-C_3_N_4_ (denoted as CN-1). The g-C_3_N_4_ ultrathin nanosheets with more hydrogen dangling bonds (denoted as CN-4) are obtained by liquid exfoliation of protonated bulk g-C_3_N_4_ (denoted as CN-2). The hydrogen content of CN-4 is higher than that of CN-3, which was confirmed by elemental analysis (Table S1†). Subsequently, systematic characterizations were performed to verify that CN-4 was in the ultrathin nanosheet structure and the C–N framework was still maintained. The colloidal suspension solution of CN-4 (Fig. 2a inset) could remain stable, without aggregation for several weeks, providing solid evidence for homogeneously exfoliated 2D nanosheets. The transmission electron microscopy (TEM) image of CN-4 shown in Fig. 2a reveals that the lateral diameters of CN-4 are ∼200 nm. Atomic force microscopy (AFM) also confirmed that the nanosheet diameter is ∼200 nm, which was consistent with the results from the TEM analyses. Meanwhile, the thickness of the products was measured by AFM. As can be seen from Fig. 2b and c, the thicknesses of the ultrathin nanosheets range from 2.2 nm to 3.0 nm, which indicates that the ultrathin nanosheets are composed of 6–9 CN atomic monolayers.

Structural information on CN-4 was elucidated using X-ray diffraction (XRD) and Raman spectroscopy. As shown in Fig. 2d, the XRD pattern of CN-4 only presents a (002) peak, suggesting a high orientation and ultrathin morphology of the as-exfoliated g-C_3_N_4_. In the Raman spectrum of CN-4, the peaks located at 705 cm^–1^, 755 cm^–1^, 1233 cm^–1^ and 1350 cm^–1^ match well with those in previous literature for g-C_3_N_4_, suggesting that CN-4 was composed of basic C–N atomic layers (Fig. 2e).^19,23^ Furthermore, the electron energy loss spectrum (EELS) confirms that only carbon and nitrogen elements exist in CN-4 (Fig. 2f). All the above results verified that the g-C_3_N_4_ ultrathin nanosheets with more hydrogen dangling bonds (CN-4) were successfully prepared with high quality.

**Fig. 3:**
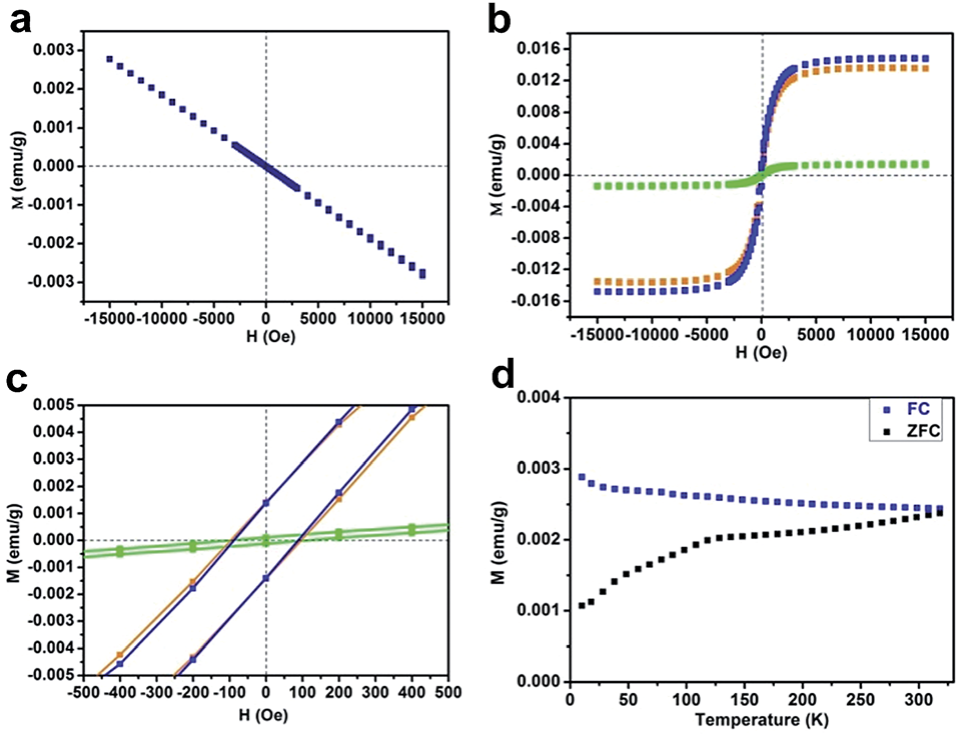
(a) *M*–*H* curves of CN-1 at 300 K. (b) *M*–*H* curves of CN-2, CN-3 and CN-4 at 300 K. The green, orange and blue curves represent CN-2, CN-3 and CN-4, respectively. (c) Enlarged central section of (b). (d) Temperature dependence of the zero field cooling (ZFC) and field cooling (FC) curves of CN-4 under a measuring field of 200 Oe. Note: *M* means magnetization and *H* means applied magnetic field.

It is well known that a substantial amino (–NH_2_) group usually exists at the edge sites of bulk g-C_3_N_4_, which is derived from thermal polycondensation of dicyandiamide.^23,24^ Considering the total energy of the hydrogen dangling bonds in different N sites of the g-C_3_N_4_ structure, the formation of hydrogen dangling bonds in the N1 sites is also attainable due to the easy promotion of more protons combining with N atoms under high power ultrasonication in aqueous solution during the exfoliation process. In order to test our expectation that hydrogen dangling bonds would bring ferromagnetic properties in the ultrathin nanosheets, a superconducting quantum interference device (SQUID) was used to investigate the magnetic properties of CN-2, CN-3, CN-4, as well as CN-1. Fig. 3a shows the magnetic field dependence of magnetization (*M*–*H*) curve of CN-1 at 300 K, clearly demonstrating that the virgin bulk g-C_3_N_4_ is diamagnetic, which implies the purity of the bulk sample. As shown in Fig. 3b and c, the expected ferromagnetic behaviors of CN-2, CN-3 and CN-4 are fully confirmed by the corresponding *M*–*H* curves, where all *M*–*H* curves exhibit saturation magnetization and a clear hysteresis loop. The saturation magnetization (*M*_s_) value of CN-4 at room temperature was as high as 0.015 emu g^–1^, with a coercivity of 87 Oe. Very weak ferromagnetism in CN-2 was understandable by the fact that only simple agitation could not provide sufficient energy to form many hydrogen dangling bonds in the N1 sites of bulk g-C_3_N_4_. In addition, the temperature dependent magnetization (*M*–*T*) curves of CN-4 presented in Fig. 3d provide further evidence proving the intrinsic room-temperature ferromagnetism of the sample. The zero field cooling (ZFC) and field cooling (FC) curves clearly show the distinct difference in the wide temperature range from 10 up to 330 K, revealing that the Curie temperature is higher than 330 K. Most importantly, there is no block temperature appearance in the ZFC curve, clearly revealing that there are no ferromagnetic clusters in our sample, and providing evidence for ferromagnetism in CN-4.^25^ Of note, ferromagnetic impurities such as Fe, Co, and Ni could also be excluded, shown by the inductively coupled plasma (ICP) results in Table S2†. Therefore, all of the above analyses confirm the inherent nature of the room temperature ferromagnetism in CN-4.

**Fig. 4:**
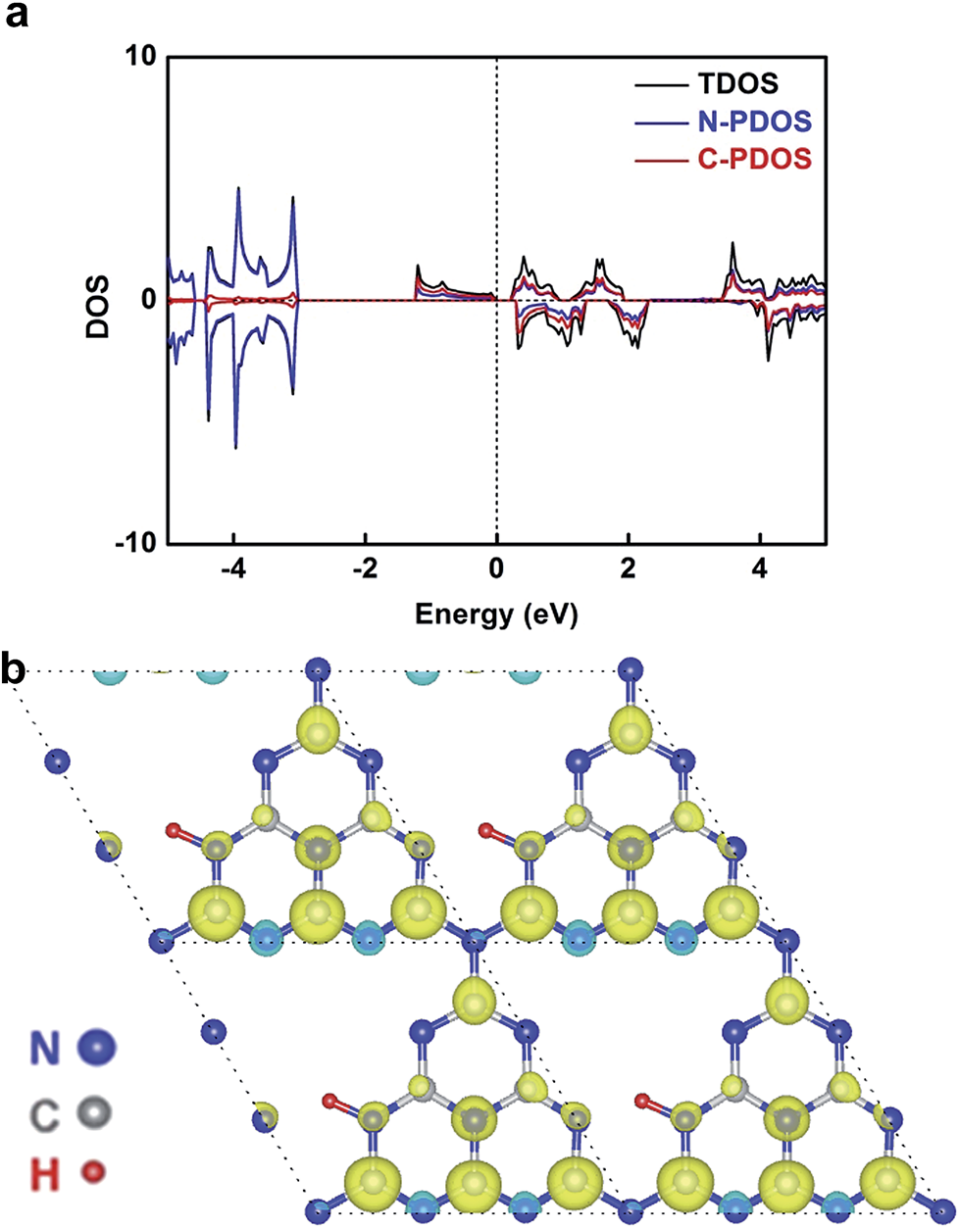
(a) TDOS and PDOS of the g-C_3_N_4_ single layer with hydrogen dangling bonds in the N1 site. The Fermi level is set at 0 eV. (b) The corresponding spin density distribution. Note: TDOS represents total density of states and PDOS represents the corresponding atomic projected density.

In our case, hydrogen dangling bonds in four samples from CN-1 to CN-4 with gradually increasing hydrogen concentrations indeed regulated the ferromagnetic behaviour. To further understand how hydrogen dangling bonds influence the ferromagnetism in CN-2, CN-3 and CN-4, single layer g-C_3_N_4_ with hydrogen dangling bonds in the N1 site was used as the calculation model to study the origin of ferromagnetism in carbon nitrides. As shown in Fig. 4a, a significant asymmetry between the spin-up state and spin-down state in the density of states (DOS) near the Fermi level suggests the intrinsic ferromagnetism of the g-C_3_N_4_ single layer with hydrogen dangling in the N1 site. As is well known, the value of the Curie temperature depends on the exchange stiffness and the DOS at the Fermi level. The calculated DOS displays obvious spin splitting at the conduction band and valence band indicating a relatively high Curie temperature in this configuration, which is identical with our experimental results. The total magnetic moments of this electronic structure are about 1.0 μB. The spin-resolved DOS projected on the p orbitals of C and N is also presented in Fig. 4a. Both of the N-PDOS and C-PDOS show significant asymmetry between the spin-up state and spin-down state near the Fermi level, illustrating that both of them contribute the magnetic moment to the total magnetic moment. Obviously, the magnetic moment in the structure is mostly attributed to the p orbital of the C atoms and the maximal magnetic moment of the C atom is about 0.15 μB. Furthermore, the spin density distribution (Fig. 4b) also indicates that the main magnetism originates from the C atoms in this structure, which is identical with the DOS. Thus, the inclusion of hydrogen dangling bonds induces intrinsic ferromagnetism in g-C_3_N_4_ ultrathin nanosheets, which is verified by both magnetic characterizations and theoretical calculations.

Controlling the concentration of hydrogen dangling bonds regulated the intrinsic ferromagnetism in the 2D carbon nitrides, with exclusion of all alternative possibilities that would arise in spin ordering. Of note, three influencing factors including heteroatom incorporation, non-metal elemental adsorption and defect are well known approaches to induce magnetism in raw non-magnetic materials. For our carbon nitrides, these three factors were not responsible for the intrinsic ferromagnetism, which was based on the detailed explanation in the following: firstly, the EELS spectrum of CN-3 and CN-4 (Fig. 2f and S9†) provided solid evidence that no heteroatoms were introduced during the sample preparation process. Therefore the ferromagnetic behavior in our sample was not triggered by heteroatom incorporation or non-metal elemental adsorption. Secondly, the molar ratios of N/C for CN-1, CN-2, CN-3 and CN-4 are all approximately 1.52, given by elemental analysis. If an abundance of N (C defect) indeed induced ferromagnetism, CN-1 would have intrinsic ferromagnetic behaviour. However, as a fact, CN-1 showed intrinsic diamagnetism shown by contrary experimental results (Fig. 3a). And thus, these analyses gave evidence to exclude the possibility that the ferromagnetism of CN-2, CN-3 and CN-4 was derived from such defects. With exclusion of the influencing factors mentioned above, the gradually increasing content of hydrogen dangling bonds in our as-obtained g-C_3_N_4_ from CN-2 to CN-4 was capable of enhancing ferromagnetism, which revealed that the hydrogen dangling bonds were able to trigger spin regulation. Indeed, magnetic coupling modulation in metal-free ultrathin nanosheets would be a significant step for future electronics and spintronics.

## Conclusions

In summary, we have demonstrated that the introduction of hydrogen dangling bonds could become a new strategy to regulate the magnetic properties of 2D metal-free systems. G-C_3_N_4_ ultrathin nanosheets with hydrogen dangling bonds, as a new metal-free room temperature ferromagnetic 2D nanomaterial, have also been confirmed for the first time. The saturation magnetization value of the g-C_3_N_4_ ultrathin nanosheets at room temperature was as high as 0.015 emu g^–1^. The 2D metal-free g-C_3_N_4_ ultrathin nanosheets with intrinsic room temperature ferromagnetism, which could carry spin-related information, are highly desirable as a building block for constructing the next generation electronic and spintronic devices. This work will broaden our horizon for achieving magnetic couple modulation in metal-free materials. Meanwhile, we anticipate that the introduction of a hydrogen dangling bond strategy could be an effective way to engineer the intrinsic physicochemical properties in 2D nanomaterials.
