# Supplementary material for: Hydrogen dangling bonds induce ferromagnetism in two-dimensional metal-free graphitic-C3N4 nanosheets
Source: Chem Sci. 2014 Oct 1;6(1):283–7. doi: 10.1039/c4sc02576h (PMC5435869; doi:10.1039/c4sc02576h)
Supplement: Supplementary file 1 [file SC-006-C4SC02576H-s001.pdf]

Electronic Supplementary Material (ESI) for Chemical Science.
This journal is © The Royal Society of Chemistry 2014

Electronic Supplementary Information for

Hydrogen dangling bonds induce ferromagnetism in two-dimensional metal-free graphitic-C_3N_4 nanosheets

Kun Xu,[a] Xiuling Li,[b] Pengzuo Chen,[a] Dan Zhou,[a] Changzheng Wu,*[a] Yuqiao Guo,[a] Lidong Zhang,[c] Jiyin Zhao,[a] Xiaojun Wu,[ab] and Yi Xie[a]

[a] Hefei National Laboratory for Physical Sciences at Microscale, University of Science and Technology of China, Hefei, 230026, P. R. China. E-mail: czwu@ustc.edu.cn

[b] CAS Key Laboratory of Materials for Energy Conversion and Depart of Material Science and Engineering, University of Science and Technology of China, Hefei, 230026, P. R. China

[c] National Synchrotron Radiation Laboratory, University of Science and Technology of China, Hefei, 230026, P. R. China

Table of contens

S1. Experimental section 3
S2. The DOS of the single layer g-C_3N_4 with hydrogen dangling bonds in different N sites..4
S3. HRTEM image of the synthetic g-C_3N_4 ultrathin nanosheets with more hydrogen dangling bonds 5
S4. FT-IR spectrum of synthetic g-C_3N_4 ultrathin nanosheets with more hydrogen dangling bonds 5
S5. XRD pattern of the synthetic bulk g-C_3N_4..6
S6. Raman spectra of the synthetic bulk g-C_3N_4 6
S7. HRTEM image of the synthetic bulk g-C_3N_4...7
S8. XRD pattern of the synthetic proton- functionalized bulk g-C_3N_4............7
S9. Characterization of synthetic g-C_3N_4 ultrathin nanosheets with hydrogen dangling bonds..8
S10. Test of room temperature ferromagnetism of synthetic g-C_3N_4 ultrathin nanosheets with more hydrogen dangling bonds against environmental viriation 9
Table S1. Hydrogen content of as-obtained samples CN-1, CN2, CN-3 and CN-4...9
Table S2. ICP results of the CN-1, CN-2, CN-3 and CN-4 10

S1. Experimental section

Preparation of bulk and proton-functionalized g-C_3N_4: The bulk and proton-functionalized g-C_3N_4 were obtained using a modified method which was reported by previous literature.[1,2] In detail, bulk g-C_3N_4 (**denoted as CN-1**) was synthesized by heating 10 g dicyandiamide at 600 ℃ for 4 h. 1 g bulk g-C_3N_4 was added to 50 ml of 10 M HCl solution and vigorous stirring for 2 h. Then, the proton-functionalized g-C_3N_4 (**denoted as CN-2**) was obtained and washed several times with distilled water to remove the HCl completely for further use.

Preparation of g-C_3N_4 ultrathin nanosheets with hydrogen dangling bonds and g-C_3N_4 ultrathin nanosheets with more hydrogen dangling bonds: 50 mg of bulk g-C_3N_4 powder was added in 100 ml distilled water and ultrasonically treated in ice water for 10 h. Subsequently, the formed suspension was then centrifuge at about 3500 rmp to remove the unexfoliated bulk sample. Finally, the g-C_3N_4 ultrathin nanosheets with hydrogen dangling bonds (**denoted as CN-3**) was obtained by centrifuging the supernatant at 14000 rpm. The synthesis process of g-C_3N_4 ultrathin nanosheets with more hydrogen dangling bonds (**denoted as CN-4**) was same to that of synthesis of g-C_3N_4 ultrathin nanosheets with hydrogen dangling bonds which was only use proton-functionalized g-C_3N_4 instead of bulk g-C_3N_4.

Characterizations. X-ray powder diffraction (XRD) was performed by using a Philips X'Pert Pro Super diffractometer with Cu Kα radiation (λ=1.54178 Å). Raman spectra were recorded at ambient temperature with LABRAM-HR Confocal Laser Micro Raman Spectrometer 750K with a laser power of 0.5mW. The transmission electron microscopy (TEM) images, was carried out on a JEM-2100F field emission electron microscope at an acceleration voltage of 200 kV. The Fourier transform infrared (FT-IR) experiment was operated on a Magna-IR 750 FT-IR spectrometer in a KBr pellet, scanning from 4000 to 400 cm^{-1} at room temperature. Atomic force microscopy (AFM) images were carried out on a DI Innova scanning probe microscope. Elemental analysis was performed on Vario MICRO. The magnetic measurement was carried out with a superconducting quantum interference device magnetometer (SQUID, quantum design MPMS XL-7).

Calculation Method. The theoretical calculations are carried using density-functional theory (DFT)[3,4] as implemented in the VASP package.[5,6] The generalized-gradient approximation (GGA) in the Perdew-Burke-Ernzerhof (PBE) form[7] and the projector augmented wave (PAW) approach[8] are employed for this spin-unrestricted DFT calculations. The cutoff energy for the palne wave is 500 eV. The thickness of vacuum space is set to 15Å. During geometric optimization, the convergence criterions for energy and force on each atom are 1.0×10-5 eV and 0.01 eV/Å, respectively. 8×8×1 Monhkorst-Pack k-points is set for the optimization.[9] A denser

k-points grid of 14×14×1 is used for the electronic structure calculations as well. Due to the DFT-PBE method always underestimates the band gap of semiconductor, the HSE06 screened hybrid DFT method is used to preferably evaluate the electronic structure.[10,11]

S2. The DOS of the single layer g-C_3N_4 with hydrogen dangling bonds in different N sites

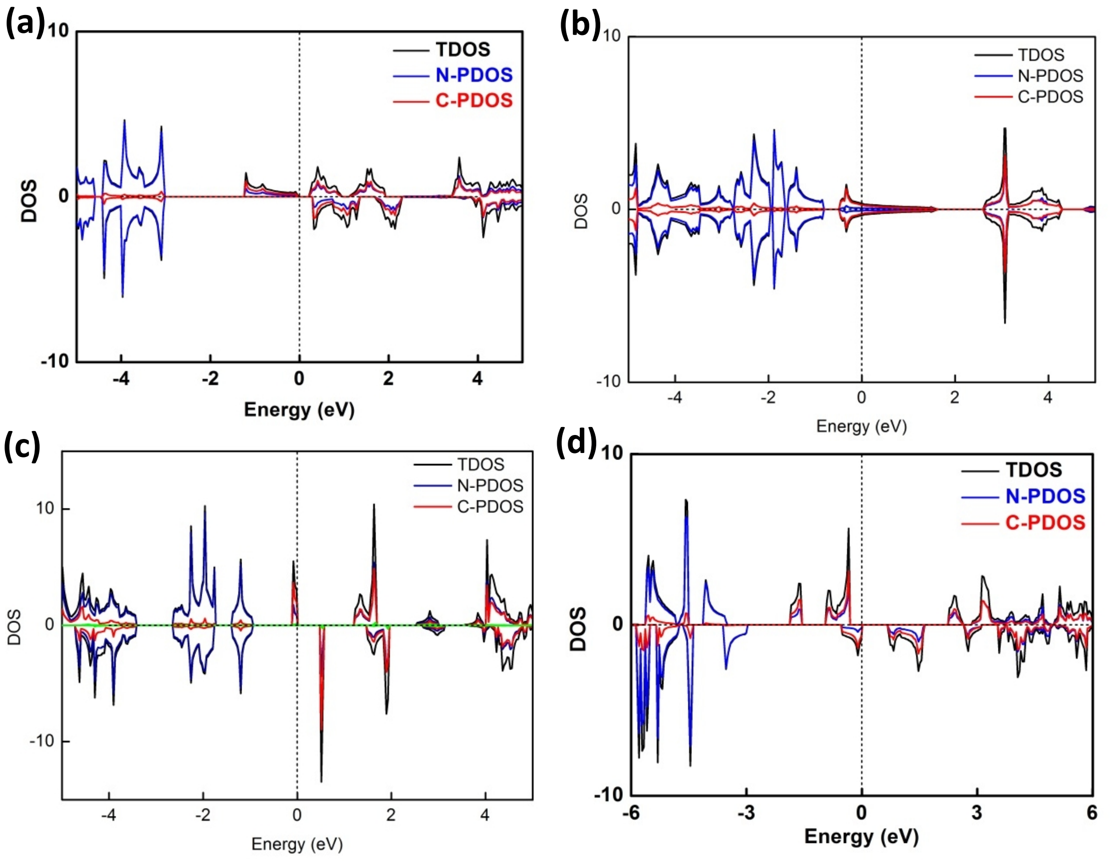

Figure S1. (a) TDOS and corresponding PDOS of g-C_3N_4 single layer with hydrogen dangling bonds in N1 site based DFT-HSE06 method. (b) TDOS and corresponding PDOS of g-C_3N_4 single layer with hydrogen dangling bonds in N2 site based DFT-HSE06 method. (c) TDOS and corresponding PDOS of g-C_3N_4 single layer with hydrogen dangling bonds in N3 site based DFT-HSE06 method. (d) TDOS and corresponding PDOS of g-C_3N_4 single layer with hydrogen dangling bonds in N1 and

N3 site based DFT-HSE06 method. The Fermi level is set at 0 eV.

S3. HRTEM image of the synthetic g-C_3N_4 ultrathin nanosheets with more hydrogen dangling bonds

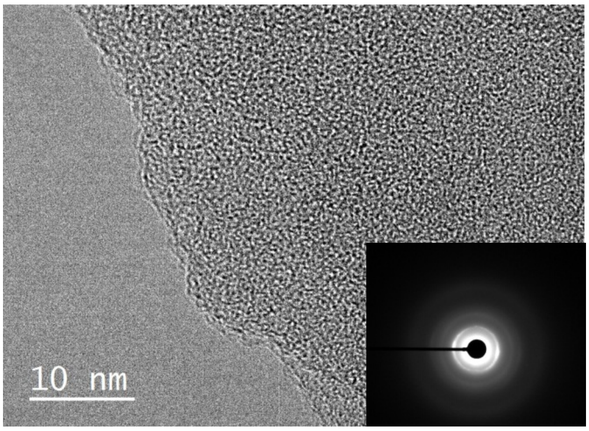

Figure S2. HETEM image of sample CN-4. Inset: the corresponding electron diffraction pattern.

S4. FT-IR spectrum of synthetic g-C_3N_4 ultrathin nanosheets with more hydrogen dangling bonds

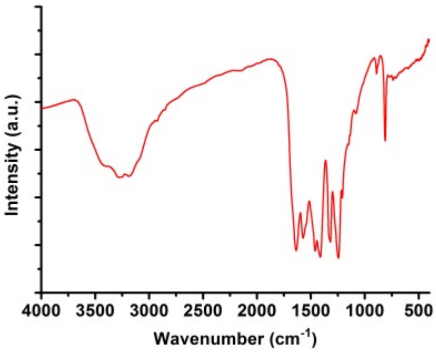

Figure S3. FT-IR spectrum of sample CN-4.

The typical FT-IR spectrum of synthetic g-C_3N_4 ultrathin nanosheets with more hydrogen dangling bonds was presented in Figure S3 which is identical with previous literature.[12-14] The absorption peak which located at 810 cm^{-1} is due to the breathing of the tri-s-triazine units. The absorption peaks of 1248, 1323, 1420, 1448, 1575, and 1645 cm^{-1} are corresponded to the typical stretching vibration of aromatic CN heterocycles. The broad bands range from 3000 to 3300 cm^{-1}

for hydrogen-bonding interactions which were aroused by secondary and primary amines.

S5. XRD pattern of the synthetic bulk g-C_3N_4

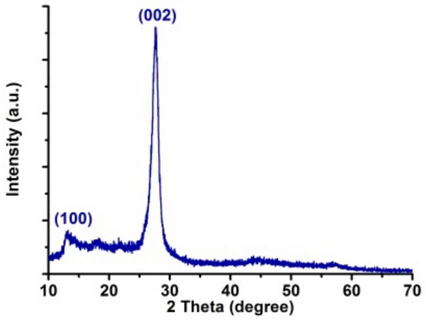

Figure S4. XRD pattern of sample CN-1.

S6. Raman spectra of the synthetic bulk g-C_3N_4

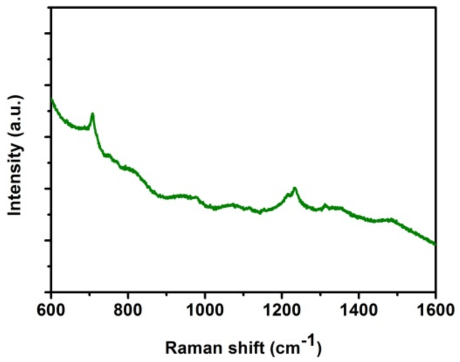

Figure S5. Raman spectra of sample CN-1.

S7. HRTEM image of the synthetic bulk g-C_3N_4

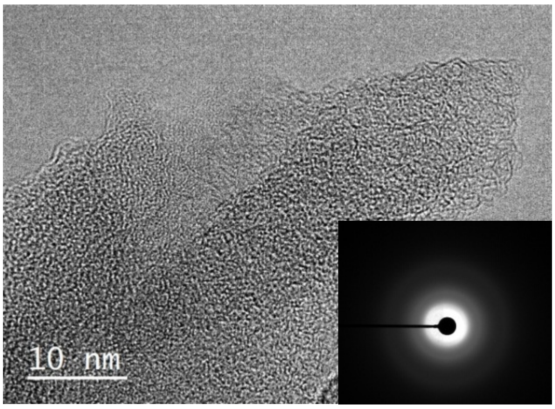

Figure S6. HETEM image of sample CN-1. Inset: the corresponding electron diffraction pattern.

S8. XRD pattern of the synthetic proton- functionalized bulk g-C_3N_4

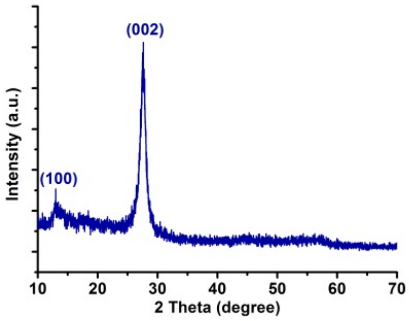

Figure S7. XRD pattern of sample CN-2.

S9. Characterization of synthetic g-C_3N_4 ultrathin nanosheets with hydrogen dangling bonds

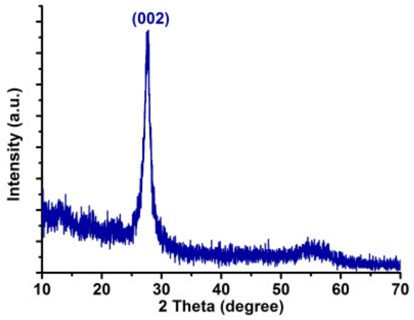

Figure S8. XRD pattern of sample CN-3.

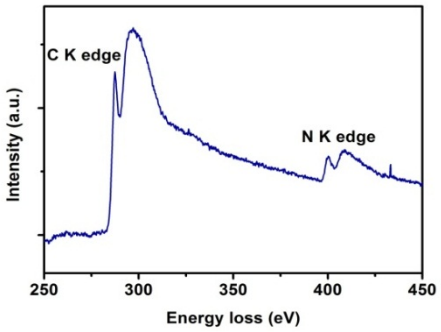

Figure S9. Electron energy loss spectra (EELS) of sample CN-3.

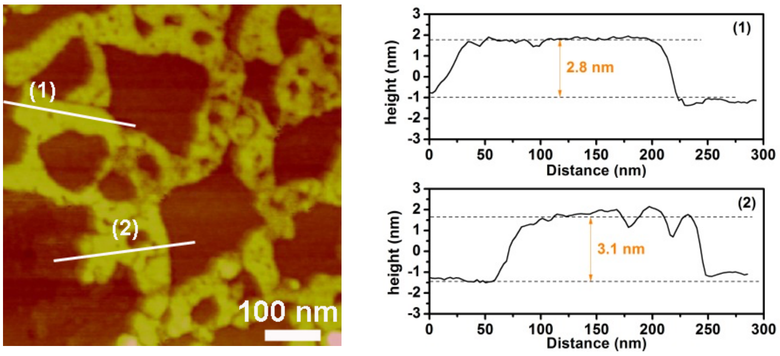

Figure S10. AFM image of sample CN-3.

S10. Test of room temperature ferromagnetism of synthetic g-C_3N_4 ultrathin nanosheets with more hydrogen dangling bonds against environmental viriation

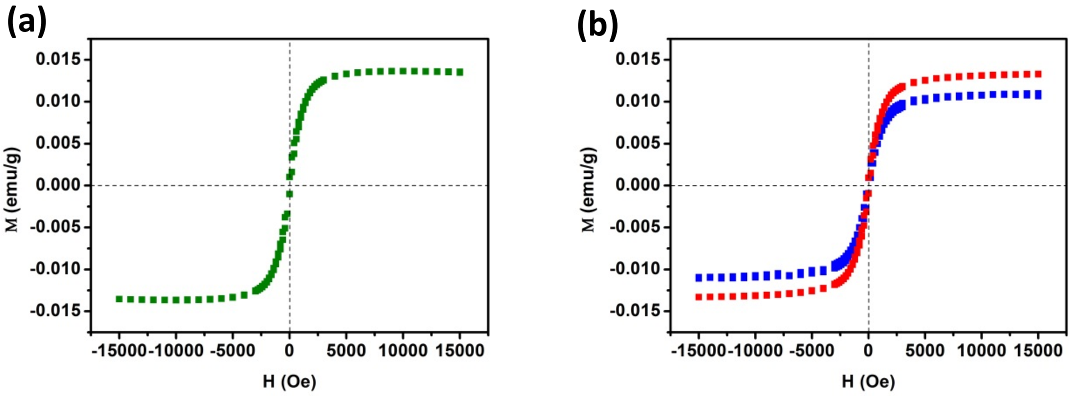

Figure S11. M-H curves of the CN-4 at 300K after three months of aging in air. (b) M-H curves of the CN-4 at 300K after heat treatment at different temperatures in argon atmosphere for 1h. Red represent at 373 K and blue represent at 473 K.

Table S1. Hydrogen content of as-obtained samples CN-1, CN2, CN-3 and CN-4

	CN-1	CN-2	CN-3	CN-4
wt %	1.99 %	2.35 %	2.41 %	2.43 %
atom %	21.30%	24.99%	25.87%	26.52%

Table S2. ICP results of the CN-1, CN-2, CN-3 and CN-4

	Fe (ppm)	Co (ppm)	Ni (ppm)
CN-1	5.6	N	N
CN-2	8.2	N	N
CN-3	8.8	N	N
CN-4	7.4	N	N

Note: N represent below the detecting limit.

Reference:

[1] X. Wang, K. Maeda, A. Thomas, K. Takanabe, G. Xin, J. M. Carlsson, K. Domen, and M. Antonietti, *Nat. Mater.*, 2009, **8**, 76-80.

[2] T. Y. Ma, Y. Tang, S. Dai, and S. Z. Qiao, *Small*, 2014, **10**, 2382-2389.

[3] P. Hohenberg, and W. Kohn, *Phys. Rev.*, 1964, **136**, B864-B871.

[4] W. Kohn, L. and J. Sham, *Phys. Rev.*, 1965, **140**, A1133-A1138.

[5] G. Kresse, and J. Furthmüller, *Phys. Rev. B*, 1996, **54**, 11169-11186.

[6] G. Kresse, and D. Joubert, *Phys. Rev. B*, 1999, **59**, 1758-1775.

[7] J. P. Perdew, K. Burke, and M. Ernzerhof, *Phys. Rev. Lett.*, 1996, **77**, 3865-3868.

[8] P. E. Blöchl, *Phys. Rev. B*, 1994, **50**, 17953-17979.

[9] H. J. Monkhorst, and J. D. Pack, *Phys. Rev. B*, 1976, **13**, 5188-5192.

[10] J. Heyd, G. E. Scuseria, and M. Ernzerhof, *J. Chem. Phys.*, 2003, **118**, 8207-8215.

[11] J. Heyd, G. E. Scuseria, and M. Ernzerhof, *J. Chem. Phys.*, 2006, **124**, 219906.

[12] Y. Shi, S. Jiang, K. Zhou, C. Bao, B. Yu, X. Qian, B. Wang, N. Hong, P. Wen, Z. Gui, Y. Hu, and R. K. K. Yuen, *ACS Appl. Mater. Interfaces*, 2013, **6**, 429-437.

[13] P. Niu, L. Zhang, G. Liu, and H.-M. Cheng, *Adv. Funct. Mater.*, 2012, **22**, 4763-4770.

[14] H. Xu, J. Yan, X. She, L. Xu, J. Xia, Y. Xu, Y. Song, L. Huang, and H. Li, *Nanoscale*, 2014, **6**, 1406-1415.
